# Review to Elucidate the Correlation between Cuproptosis-Related Genes and Immune Infiltration for Enhancing the Detection and Treatment of Cervical Cancer

**DOI:** 10.3390/ijms251910604

**Published:** 2024-10-01

**Authors:** Pratibha Pandey, Seema Ramniwas, Shivam Pandey, Sorabh Lakhanpal, G. Padmapriya, Shivang Mishra, Mandeep Kaur, Ayash Ashraf, M Ravi Kumar, Fahad Khan

**Affiliations:** 1 Post Doctoral Department, Eudoxia Research University, New Castle, DE 19808, USA; shukla.pratibha1985@gmail.com; 2 Centre for Research Impact and Outcome, Chitkara University Institute of Engineering and Technology, Chitkara University, Rajpura 140401, India; 3 University Centre for Research and Development, Chandigarh University, Gharuan, Mohali 140413, India; seema.ramniwas@gmail.com; 4 School of Applied and Life Sciences, Uttaranchal University, Dehradun 248007, India; pandeyshivam547@gmail.com; 5 School of Pharmaceutical Sciences, Lovely Professional University, Phagwara 144411, India; sorabh.lakhanpal@lpu.co.in; 6 Department of Chemistry and Biochemistry, School of Sciences, JAIN Deemed to be University, Bangalore 560069, India; g.padmapriya@jainuniversity.ac.in; 7 NIMS Institute of Pharmacy, NIMS University Rajasthan, Jaipur 303121, India; shivang.mishra@nimsuniversity.org; 8 Department of Sciences, Vivekananda Global University, Jaipur 303012, India; mkphd2024@gmail.com; 9 Chandigarh Pharmacy College, Chandigarh Group of College, Jhanjeri, Mohali 140307, India; ayash2810.research@cgcjhanjeri.in; 10 Department of Chemistry, Raghu Engineering College, Visakhapatnam 531162, India; mravikumar.chemistry@outlook.com; 11 Center for Global Health Research Saveetha Medical College, Saveetha Institute of Medical and Technical Sciences, Chennai 600077, India

**Keywords:** copper, cuproptosis, cervical cancer, immunotherapy, metabolism, long coding RNAs

## Abstract

Copper is a vital trace element in oxidized and reduced forms. It plays crucial roles in numerous biological events such as redox chemistry, enzymatic reactions, mitochondrial respiration, iron metabolism, autophagy, and immune modulation. Maintaining the balance of copper in the body is essential because its deficiency and excess can be harmful. Abnormal copper metabolism has a two-fold impact on the development of tumors and cancer treatment. Cuproptosis is a form of cell death that occurs when there is excessive copper in the body, leading to proteotoxic stress and the activation of a specific pathway in the mitochondria. Research has been conducted on the advantageous role of copper ionophores and chelators in cancer management. This review presents recent progress in understanding copper metabolism, cuproptosis, and the molecular mechanisms involved in using copper for targeted therapy in cervical cancer. Integrating trace metals and minerals into nanoparticulate systems is a promising approach for controlling invasive tumors. Therefore, we have also included a concise overview of copper nanoformulations targeting cervical cancer cells. This review offers comprehensive insights into the correlation between cuproptosis-related genes and immune infiltration, as well as the prognosis of cervical cancer. These findings can be valuable for developing advanced clinical tools to enhance the detection and treatment of cervical cancer.

## 1. Introduction

Cuproptosis causes proteotoxic stress, which ultimately results in cellular damage. Copper (Cu), a vital micronutrient, regulates various fundamental processes in all living organisms, including respiration, cell growth, and neurotransmitter production [1]. Fluctuations in trace element concentrations are essential for the development of cervical cancer [2]. Although trace elements are found in small quantities in the human circulatory system, they significantly impact several biochemical and enzymatic events. These reactions play a significant role in the development of various illnesses and severe forms of cancer [3].

Furthermore, malignant cells require trace minerals for their survival. The presence and activity of trace elements, specifically copper (Cu), zinc (Zn), selenium (Se), iron (Fe), arsenic (As), cadmium (Cd), and manganese (Mn), have significant effects on the development, occurrence, growth, and reduction of tumors [4]. Trace elements have demonstrated considerable potential and will, therefore, expand novel therapeutic strategies for diagnosing and treating cervical cancer, whether caused by HPV or non-HPV factors. The high redox activity of copper poses a dual threat to cell viability, and irregular copper metabolism is frequently linked to cancer and other illnesses [5]. The ratio of serum copper (Cu) to zinc (Zn) is a significant factor in determining the occurrence of malignant gynecological tumors and the stage of cervical cancer [6]. The variability in the quantities of trace elements within and outside cells makes them a promising choice for detecting and treating cervical cancer.

This review extensively evaluates the prominent field of copper biology and its applications in cancer treatment. It focuses explicitly on copper homeostasis and cuproptosis to aid the development of advanced inorganic anticancer medications. Consequently, deactivation of specific tumor suppressor genes may result in the onset and progression of carcinogenesis [7]. This increase in Cu levels may be attributed to the transfer of Cu from the tissue to the serum. Copper (Cu) has a significant role in the development of cancer, which may be related to its ability to engage with specific proteins and facilitate cellular proliferation through the activation of angiogenic growth factors. Increased concentrations of Cu led to the initiation of angiogenesis, thereby augmenting the vascularization necessary for tumor proliferation [8]. Oxidative stress dysregulation hampers cellular DNA repair mechanisms by excessively generating reactive oxygen species (ROS), a significant factor in cancer development [9].

Increased blood copper levels have been documented in different forms of malignancies. In vitro, introducing Cu into DNA increases DNA base damage, leading to a higher occurrence of mutations [10]. Copper, acting as a co-factor for numerous redox enzymes, can attach to nucleic acids and proteins, potentially oxidizing proteins and lipids. Copper ions are necessary for the optimal operation of metalloenzymes such as cytochrome-C oxidase, α-amylase, carbonic anhydrase, superoxide dismutase, tyrosinase, dopamine hydroxylase, ceruloplasmin, ALA synthase, catalase, uricase, and ascorbic acid oxidase [11]. In addition, providing trace element supplements during the emergence of cervical cancer can effectively inhibit tumor growth. Copper is crucial in facilitating the rapid development and multiplication of cancer cells, called ‘cuproplasia’ [12]. Nevertheless, an excessive buildup of copper in the mitochondria also induces ‘cuproptosis’ (a novel form of programmed cell death) characterized by the instability of Fe-S cluster proteins and the aggregation of DLAT [13]. Research has shown that Cu promotes the formation of blood vessels in tumors by activating several proteins that stimulate angiogenesis, including essential FGF (fibroblast growth factor) and VEGF (vascular endothelial growth factor) [14]. Additionally, copper plays a role in transmitting signals within endothelial cells, which in turn affects angiogenesis [15].

Cervical cancer is a malignancy that explicitly targets the cervix and primarily manifests as squamous and adenocarcinoma subtypes [16]. Early-stage lymphatic metastasis is prevalent and associated with a somewhat unfavorable outcome. Therefore, identifying novel biological targets is crucial. Numerous cervical cancers are associated with HPV infection [17]. The prevalence of cervical cancer has diminished owing to the use of HPV vaccination and screening. The prognosis of cervical cancer is strongly correlated with clinical stage and pathological type. Cervical adenocarcinoma, in its early stage, has a high tendency to spread through the lymphatic system, resulting in a poorer prognosis than other gynecological cancers. The primary modalities for the treatment of cervical cancer include surgery, radiation, and chemotherapy [18]. However, radiotherapy and chemotherapy can have detrimental consequences for patients, potentially impairing their bodily function.

Furthermore, with frequent administration of radiotherapy and chemotherapy, patients may acquire drug resistance or tolerance, leading to a steady decline in the effectiveness of the treatment. Immunotherapy is administered to individuals diagnosed with cervical cancer [19]. Therefore, there is a pressing demand for new and innovative therapies for cervical cancer. Hence, it is crucial to identify reliable biomarkers for predicting therapeutic response and prognosis, as well as devising efficacious treatment approaches for individuals with cervical cancer. Studying changes in the immune microenvironment and checkpoint genes in tumors with different levels of blood vessel growth can offer essential knowledge regarding creating specific combinations of therapies that target blood vessels and enhance the immune system response.

## 2. Metabolism of Copper at Both Cellular and Organ Level

Copper homeostasis is controlled by many mechanisms, such as the absorption of copper by intestinal or tissue cells, its circulation in the blood, and its usage, excretion, or export by tissue cells [20]. The small intestine takes up copper in mammals’ diet. Divalent copper ions are found in the small intestine as extracellular copper ions. Enterocytes cannot directly absorb Cu (II) until they undergo reduction to Cu (I) via their interaction with the STEAP family of metalloreductases [21]. Copper (Cu) mainly enters the digestive tract or other somatic cells via the SLC31 copper osmosis family transporter, namely SLC31A1/CTR1 (solute carrier family 31 member 1) and SLC31A2/CTR2 (solute carrier family 31 member 2). Currently, two potential processes affect the expression of SLC31A1, which regulates copper absorption. SP1 mediates the regulation of SLC31A1 expression. Furthermore, increased levels of copper trigger endocytosis and subsequent destruction of the SLC31A1 protein. Moreover, the absorption of copper by SLC11A2/DMT1 (a member of solute carrier family 11) could serve as compensatory mechanism in cases of SLC31A1 deficiency [22]. It is crucial to ascertain whether these copper transporters in the plasma membrane have tumor-type-specific functions in facilitating copper absorption.

### 2.1. Copper at Cellular Level

After copper ions are transported into the cell, copper chaperones facilitate the distribution of copper to various cellular compartments, including the cytoplasm, mitochondria, Golgi apparatus, and nucleus, to regulate diverse cellular activities. CCS, also known as copper chaperone for superoxide dismutase, is a cytoplasmic protein that plays a role in transporting copper to particular proteins, including SOD1 or superoxide dismutase. SOD1 is an antioxidant protein found primarily in the cytoplasm, with a minor fraction present in the mitochondrial intermembrane space [23]. Aberrant SOD1 expression is intricately linked to the proliferation and maturation of cancer cells [24]. CCS controls the allocation of SOD1 between the intermembrane space and cytoplasm in a manner that depends on the presence of oxygen. This regulatory mechanism preserves the stability of reactive oxygen species (ROS) in the body and mitigates ROS production via the electron transport chain (ETC), thereby preventing the harmful effects of excessive Cu on oxidation [25].

The import of copper ions into mitochondria mainly relies on COX17, a chaperone for cytochrome c oxidase copper [26]. COX17 facilitates the transfer of Cu (I) ions from the cytoplasm to the mitochondrial membrane proteins SCO1 and SCO2. These proteins are involved in manufacturing cytochrome C oxidase 1/2, respectively [27]. The copper ions are inserted into MT-CO2/COX2, the mitochondrially encoded cytochrome c oxidase II. COX17 can transfer copper from the cytoplasm to MT-CO1/COX1, a mitochondrially encoded cytochrome c oxidase I, by providing copper to COX11, a copper chaperone for cytochrome c oxidase [28]. MT-CO1 and MT-CO2 are two subunits of complex IV that bind to copper. Complex IV promotes the transferring of electrons from CYCS (cytochrome c, somatic) and facilitates the electrochemical synthesis of adenosine triphosphate (ATP). Therefore, cellular reserves of copper are strongly connected to the process of mitochondrial oxidative phosphorylation. COX17 is required for the function of MT-CO1 and MT-CO2 in the mitochondrial respiratory chain, which has a crucial impact on tumor growth, invasion, and metastasis [29]. Hence, COX17 has promise as a viable molecular target for addressing solid tumors or hematological malignancies.

### 2.2. Copper at Organ Level

The transport of copper to the trans-Golgi apparatus can be facilitated by the copper chaperone ATOX1, also known as the antioxidant one copper chaperone. ATOX1 binds to Cu(I) in the cytoplasm and transports it to ATP7A or ATP7B, copper-dependent ATPases in the Golgi network. MEMO1, a mediator of cell motility 1, increases the affinity of Cu(I) for ATOX1, thereby inhibiting the excessive ROS (reactive oxygen species) generation caused by excess copper [30]. When copper levels increase, ATP7A and ATP7B move from the Golgi apparatus to either post-Golgi locations or lysosomes. This movement helps to remove excess copper from the cells. Increased expression of ATOX1 facilitates the ability of cancer cells to withstand the effects of genotoxic agents. The interaction between the two copper regulators, ATOX1 and ATP7A, facilitates the movement of breast cancer cells [31].

Consequently, blocking the transport of copper to the trans-Golgi is a potential approach to hinder the movement and infiltration of tumors [32]. The CCS transports copper to the nucleus, which might stimulate the transcription factor HIF1 (hypoxia-inducible factor 1). Alternatively, ATOX1 can transport copper into the nucleus and functions as a transcription factor that relies on copper. Pharmacological inhibition of CCS or ATOX1 leads to the buildup of copper and reduces tumor growth, both in vivo and in vitro [33]. Similarly, the histone H3-H4 tetramer functions as an oxidoreductase, facilitating the conversion of Cu(II) to Cu(I), thus controlling nuclear processes [34]. These advancements are partially based on foundational research in the field of copper-related techniques that disrupt gene expression in cancer by manipulating the activity of transcription factors or modifying chromosomal functions.

Unbound copper ions not attached to other molecules form reactive oxygen species (ROS) or cause cellular toxicity. However, this harmful process is prevented by proteins that capture and isolate copper ions within cells. For example, excess intracellular copper ions are stored by MTs (metallothioneins) and glutathione (GSH) [35]. Metallothioneins (MTs) are a group of small proteins that consist of clusters of metal atoms linked to thiolate groups [36]. MT1 and MT2 bind to a significant number of copper ions carried by SLC31A1, and the presence of metal ions such as copper can stimulate the expression of MT [37]. MTs are expressed in various tumor cells in diverse manners and control tumor growth, differentiation, and metastasis. GSH, or glutathione, is the predominant non-protein thiol and is likely the initial receptor for excess free copper ions before copper binding occurs through MT or metallothionein [38]. The breast cancer stem cell phenotype is influenced by the repression of copper-dependent MAP2K1/MEK1 activity caused by HIF1-induced GSH. MTs and GSH work together as a natural defensive mechanism to protect cells, especially cancer cells, from the harmful effects of Cu [39]. Nevertheless, a crucial challenge in devising ways to combat oxidative stress is the determination of the copper-independent roles of MT and GSH.

The copper-transporting ATPases ATP7A and ATP7B have a crucial function in the export of copper ions from the extracellular environment [40]. Genetic mutations in ATP7A and ATP7B lead to copper transport abnormalities known as Menkes and Wilson illnesses [41]. ATP7A and ATP7B are situated in the trans-Golgi network when copper levels are low under normal conditions [42]. When there is more copper in the body, ATP7A and ATP7B move to either the outer layer of the cell or inside small compartments. Simultaneously, ATP7A and ATP7B facilitate the transportation of copper from the trans-Golgi network to post-Golgi vesicles [43]. The vesicles containing copper can merge with the plasma membrane and release copper into the surrounding extracellular environment. Copper ions facilitate the export of copper from cells, particularly cancer cells [44].

**Figure 1:**
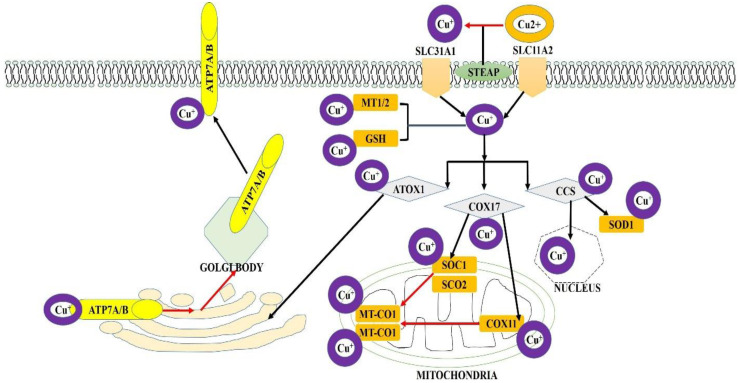
Copper metabolism is regulated at both organ and cellular levels. Copper ion uptake is mediated by SLC31A2 and SLC31A1, and copper export is driven by ATP7B and ATP7A. In cells, copper is transported to different organelles (for bioavailability) via numerous copper-binding proteins (COX17, CCS, and ATOX1). The binding of MT2, GSH, and MT2 to copper can prevent the cytotoxicity of excess copper.

Increased ATP7A expression protects against excessive copper-induced toxicity in colorectal cancer cells with a mutation in the KRAS gene. According to these findings, focusing on ATP7A is a method that can effectively eradicate colorectal cancer driven by the KRAS gene. It is worth mentioning that ATP7A and ATP7B also control the removal of platinum drugs from cells. Therefore, increased levels of ATP7A and ATP7B expression play a role in developing resistance to platinum-based chemotherapy in ovarian cancer. These findings indicate that ATP7A and ATP7B are more involved in tumor biology (Figure 1).

## 3. Crosstalk between Copper and Cancer

Copper, a vital nutrient, plays a crucial role in sustaining the activity of enzymes and the function of transcription factors. Excessive amounts of copper lead to the clumping together of lipoylated dihydrolipoamide S-acetyltransferase (DLAT), which is connected to the mitochondrial tricarboxylic acid (TCA) cycle [45]. This causes stress to the proteins and triggers a new kind of cell death called cuproptosis. Cuproptosis plays a crucial part in the advancement of cancer and is seen as a possible approach to treating cancer. The presence of copper in cells is important for life since it serves as a catalytic cofactor for crucial enzymes involved in energy conversion, iron collecting, oxygen transport, and intracellular oxidative metabolism. The cellular copper concentration is regulated by metabolic demands and fluctuations in the cellular environment [46]. Insufficient or excessive amounts of copper can significantly impact the cellular physiology of bacteria and human cells, underscoring the complexity and importance of copper in our understanding of cancer [47].

Cancer immunotherapy has received significant interest due to advancements in immune checkpoint blockage. Additionally, cuproptosis is closely linked to the regulation of antitumor immunity. Studies have indicated that copper is related to the ability to resist platinum-based anticancer drugs and can be utilized as a radiotherapeutic agent in combined cancer treatment [48]. Copper can enhance the antitumor efficacy in patients by forming a bond with disulfiram (DSF). These findings demonstrated the significant potential of copper in treating malignancies that have an inherent resistance to apoptosis. Recent research has identified the ubiquitin–proteasome system (UPS), copper deficiency-induced angiogenesis suppression in cancer cells, and cuproptosis as the main mechanisms of copper-inducing cancer cell death [49].

### 3.1. Copper and Oxidative Stress Generation

The demise of healthy or cancerous cells occurs due to oxidative stress induced by imbalanced oxidative–antioxidant equilibrium inside the body and is mainly distinguished by elevated ROS levels [50]. Copper ions (Cu^+^) in the body regulate the balance of copper by facilitating the uptake and removal of copper. An excess of copper stimulates the Fenton reaction, leading to excessive ROS generation, resulting in protein oxidation, DNA damage, nuclear damage, and impairment of mitochondria and different enzymes [51]. Copper-induced oxidative stress primarily appears in two distinct manners. ROS can directly oxidize and break down specific copper complexes through the Fenton reaction [52]. This process results in necrotic apoptosis and toxic harm to cancer cells. Copper facilitates the production of highly reactive hydroxyl radicals (-OH), leading to increased levels of reactive oxygen species (ROS) in cancer cells and subsequent cancer cell death [53]. Copper can exhaust the antioxidant glutathione (GSH) by converting reduced GSH into oxidized glutathione disulfide (GSSG). This process disturbs the GSH-related antioxidant defense system and diminishes the system’s capacity to eliminate highly reactive -OH, ultimately leading to the death of cancer cells. Copper complexes can trigger apoptosis and autophagy by disrupting mitochondrial function due to oxidative stress, as the excessive ROS produced by copper is also detrimental to mitochondria. Studies have shown that elesclomol (ES), a copper chelator with high lipid solubility, binds to extracellular Cu^2+^ and creates a complex called ES-Cu^2+^. This complex helps transport copper to mitochondria for redox reactions and induces oxidative stress. As a result, cancer cells experience apoptosis [54].

### 3.2. Copper and Angiogenesis Inhibition

Malignant angiogenesis promotes the growth, invasion, and spread of cancer cells. This is because neoangiogenesis (formation of new blood vessels) is the initial step in the proliferation and spread of cancer [55]. Copper is the primary factor in promoting angiogenesis, as it directly stimulates the migration and proliferation of endothelial cells along with fibronectin formation [8]. Hypoxia-inducible factor (HIF-1) can attach to copper, stimulating crucial components that regulate angiogenesis [56]. Angiogenin is a chemical that binds to copper, which activates endothelial cells to commence the process of angiogenesis. Copper deficiency inhibits the activation of the angiogenic switch, halts the proliferation of endothelial cells, and disrupts the cell cycle [57]. Copper depletion is a unique therapeutic strategy employed in cancer treatment to hinder the formation of new blood vessels that supply tumors, a process known as cancer angiogenesis [58]. Tetrathiomolybdate (TTM) is a compound currently gaining significant interest in cancer therapy due to its ability to suppress cancer angiogenesis. It achieves this by interacting with copper ions to produce insoluble copper–molybdenum–sulfur clusters. TTM has demonstrated significant potential as an adjuvant when used with anticancer medicines [59]. Given that copper-mediated anti-angiogenic effects can hinder the blood vessel growth necessary for cancer growth and spread, as well as modify the cancer immune microenvironment, immune checkpoint inhibitors (ICI) can facilitate the normalization of blood vessels. Consequently, combining immunotherapy with copper complexes is a promising anticancer therapy approach. This combination is crucial in suppressing cancer cell proliferation and triggering cell death [60].

### 3.3. Copper and Ubiquitin Proteasome System

The ubiquitin–proteasome system (UPS) is one of the processes responsible for the degradation of many proteins in the human body [61]. The UPS plays a crucial role in the proliferation of cancer cells, the process of cell death (apoptosis), the formation of new blood vessels (angiogenesis), and metastasis [62]. Studies have shown that Cu^2+^ can hinder the proteasome by directly attaching to it. Disulfiram (DSF) and other copper complexes have been used as proteasome inhibitors in cancer treatment. DSF, a highly promising medicine for treating cancer, functions as an acetaldehyde dehydrogenase inhibitor. It achieves this by binding to Cu^2+^ ions, effectively eliminating cancer cells [63]. Diethyldithiocarbomate (DDTC) is rapidly synthesized in the body from DSF. Recent findings reveal that DDTC can create a dinuclear complex with Cu^+^ or DDTC-Cu^+^ [64]. This copper complex leads to the buildup of ubiquitinated proteins, an elevation in p27 levels, and the suppression of nuclear factor-kappa B (NF-κB) expression [65]. Consequently, this inhibits the proliferation of cancer cells and the functioning of the proteasome, both in laboratory settings and within living organisms. NF-κB, being a pivotal transcription factor, plays a critical role in cell proliferation, invasion, spread to other parts of the body, and the formation of new blood vessels. The presence of Cu^2+^ significantly reduces cancer cells’ resistance to bortezomib, enhancing its effectiveness in fighting cancer [66]. Using copper-complex-targeted ubiquitin–proteasome inhibitor medication offers a novel approach to conventional anticancer therapy.

## 4. Copper and Cervical Cancer

Much research has investigated the correlation between serum copper levels and cervical cancer. These studies have consistently found strong evidence indicating that patients with cervical cancer had higher serum copper levels compared to control groups. This suggests that exposure to serum copper is a risk factor for developing cervical cancer [67]. This study assessed the cytotoxic efficacy of a newly developed copper (II) complex (LQM402) on cervical cancer cell lines. The results revealed that LQM402 demonstrated specific cytotoxicity against HeLa and Ca Ski cells. The chemical LQM402 shows potential as a safe and effective treatment for cervical cancer [68]. Tumor resistance is a worldwide obstacle in the field of tumor therapy. Disulfiram exhibited cytotoxicity against cervical cancer cell lines in a manner that relied on the presence of copper (Cu). The disulfiram/copper (DSF/Cu) compound disrupted the S-phase and suppressed the expression of stemness markers in cervical cancer cells. In addition, DSF/Cu can decrease the population of cancer stem cell-like LGR5+ cells, which are responsible for causing cisplatin resistance in cervical cancer cells. The DSF/Cu combination exhibited superior antitumor effectiveness against cervical cancer compared to both in vitro and in vivo cisplatin. Hence, the DSF/Cu complex could serve as a promising therapeutic approach for individuals with cervical cancer [69].

Nanotechnology is highly beneficial for numerous sectors, particularly medicine, where it plays a significant role [70]. The wide-ranging use of inorganic metals in cancer therapy can be attributed to their many biological characteristics not present in organic substances [71]. Copper is particularly intriguing because of its exceptional stability and cost-effectiveness, among other compounds. Metallic nanoparticles have garnered significant attention across various sectors due to their small size, large surface area, optical and chemical characteristics, and high conductivity. The copper nanoparticles exhibited distinct antibacterial, antioxidant, and anticancer properties [72]. Various techniques have been employed to synthesize CuO NPs, such as sol–gel, electrochemical, thermal decomposition, microwave irradiation, solid-state reactions, precipitation, solution combustion, ultrasonic mixing, self-assembly, and the hydrothermal route with PEG [73]. These techniques include elevated temperatures, increased pressure, and dangerous chemicals. Additionally, certain harmful substances that are adsorbed onto the surface of nanoparticles can lead to adverse health effects.

Researchers aimed to harness nanotechnology to develop a drug combining traditional herbal medicine. This approach was chosen because nanodrugs have a higher level of precision in targeting specific areas and enhance the effectiveness of conventional medicine [74]. Consequently, a copper nanodrug was created using an extract from the plant H. cordata (Hc-CuONPs), and its efficacy against cervical cancer cells was evaluated. The Hc-CuONPs produced using green methods had strong lethal effects on HeLa cells, a type of human cervical cancer cells, via triggering apoptosis and modifying the PI3k/AKT/mTOR signaling pathway. The apoptotic alterations were thoroughly documented and confirmed by the upregulation of pro-apoptotic proteins Bax and Bad and the downregulation of anti-apoptotic proteins Bcl2 and Bcl-xl. This study yielded valuable insights into the cytotoxic causes and apoptosis route by utilizing Hc-CuONPs against HeLa cells [75]. Therefore, the green-produced Hc-CuONPs can potentially be a potent anticancer agent for treating cervical cancer.

A novel copper (II) complex with [Cu(L)(phen)]MeOH (L = 4-chloro-2-[(2-hydroxyphenyl)iminomethyl]phenol) mixed ligands was successfully produced. The compound is classified under the orthorhombic crystal system, and five ligands coordinate the Cu(II) ion in a highly deformed square pyramidal geometry. This study demonstrated that the complex could trigger apoptosis in a manner that was dependent on the dosage. Additionally, it was found to be somewhat linked to the arrest of the cell cycle. Consistent with the findings of the DNA cleavage experiment, the comet assay revealed that the complex caused significant DNA fragmentation. These research findings demonstrated a positive correlation between the concentration of the complex and the generation of reactive oxygen species. The study proposed that the complex can induce programmed cell death in HeLa cells by activating a pathway that causes oxidative damage to DNA [76].

Cu_4_O_3_, a copper oxide that has received less attention than other copper oxides, holds significant promise in cancer treatment, particularly in its nanoform [77]. Our investigation aimed to produce Cu_4_O_3_ nanoparticles (NPs) using a water-based extract from pumpkin seeds and evaluated their efficacies in inhibiting the growth of cervical cells. Cells treated with Cu_4_O_3_ NPs showed a substantial increase (>3.5-fold) in reactive oxygen species formation, as well as changes in MMP and a decrease in cell adhesion/migration. Our study demonstrated the remarkable potential of Cu_4_O_3_ nanoparticles in inhibiting the growth of cervical cancer cells. However, further research is needed to validate these findings in an appropriate in vivo model. We also propose the use of a green-based, eco-friendly, and cost-effective approach for creating new nanoformulations, based on our findings [78].

Utilizing copper-specific chelators to target copper in cancer cells can be a successful technique for fighting cancer [79]. A tetrazole derivative (ligand-L) was produced based on the nucleus of pregnenolone acetate, which acts as a copper chelator [80]. This derivative demonstrated notable cytotoxic action against C33A cervical cancer cells. The cytotoxicity of ligand-L is caused by the redox cycling of copper, which generates reactive oxygen species (ROS) that result in DNA damage and apoptosis. A novel tetrazole derivative, derived from pregnenolone acetate, has been synthesized and tested against C33A cells. This compound explicitly targets cellular copper and effectively induces pro-oxidant death in cancer cells. This research offers valuable knowledge regarding the creation of novel chemical compounds that possess enhanced abilities to bind to copper and promote oxidative stress in cancer cells [81].

Managing the intricacies of cancer is a continuous struggle in the field of medical treatment. Nanomedicine has carved out a specialized area in gene therapy where the use of inorganic nanoparticles has become appealing for delivering genes effectively [82]. Copper oxide nanoparticles (CuONPs) have received the least research attention among the many metallic nanoparticles in gene transfer [83,84]. Copper oxide nanoparticles (CuONPs) were produced through a biological process utilizing an extract from the leaves of Melia azedarach. The nanoparticles were then modified using chitosan and polyethylene glycol (PEG) and finally linked to the targeted molecule folate. The nanoparticles demonstrated remarkable affinity and safeguarding of the reporter gene, pCMV-Luc-DNA. The in vitro cytotoxicity experiments showed that the cell viability was above 70% in human embryonic kidney (HEK293), breast adenocarcinoma (MCF-7), and cervical cancer (HeLa) cells. Additionally, a notable transgene expression was observed by the luciferase reporter gene assay. These nanoparticles generally exhibited positive characteristics and effective gene transport, indicating their prospective use in gene therapy [85].

Metal oxide nanoparticles function as carrier matrices for compounds of biological significance [86]. TiO_2_ nanoparticles were linked with various copper complexes using the sol–gel technique [87]. Their biological activity was assessed by measuring DNA degradation and evaluating their cytotoxic effect on HeLa cells. The Cu/TiO_2_ nanoparticles initiated the degradation of DNA after 15 min. The study by González-García et al. (2020) demonstrated that these nanoparticles have potential for use in the treatment of uterine-cervical cancer [88].

A single crystal of the copper complex is synthesized using NaSCN and identified as CuL (SCN). The copper complex CuL (SCN) is used to investigate its effectiveness as a medicinal agent [89]. The compound exhibits cytotoxicity towards high-risk HPV-positive cervical cancer cells (SiHa and HeLa). Additionally, it effectively generates and accumulates reactive oxygen species (ROS). The complex also triggers nuclear blebbing and exhibits DNA degradation, as evidenced by the DNA laddering experiment [90]. This study investigates using phytoconstituents for environmentally friendly production of copper oxide (CuO) nanoparticles (NPs) as a potential alternative to standard chemical synthesis methods known to have adverse environmental effects. The synthesized nanoparticles displayed a spherical shape and showed notable toxicity against HeLa cells. The CuO nanoparticles exhibited remarkable photocatalytic efficacy, resulting in a 91.12% degradation of the organic dye methylene blue during a 180 min timeframe under UV irradiation. This indicates their potential for effectively decomposing organic contaminants in the environment. This study highlights the environmentally friendly production of copper oxide nanoparticles (CuO NPs), which have the potential to significantly influence energy, the environment, and medicine using sustainable methods [91].

Copper oxide nanoparticles (CuONPs) were produced using an environmentally friendly method that employed a water-based extract from black beans. The levels of reactive oxygen species (ROS) originating from mitochondria were elevated, leading to the oxidation of lipids in the liposomal membrane. This process plays a role in controlling several signaling pathways and affecting the motions of cells during cytokinesis. The mitochondrial fragmentation disruption assay verified the modification in the structure of the mitochondria following the incubation with nanoparticles. Furthermore, the clonogenic assay proved that cancer cells treated with NPs could not increase effectively. The experimental findings demonstrate that the CuO nanoparticles can trigger apoptosis and inhibit the growth of HeLa cells [92]. A minor release of Cu and Fe ions from the CuFe nanoparticles in an acidic environment was seen, offering direct proof of the degradability of CuFe nanoparticles without the potential for long-term retention in the body. Furthermore, the exceptional photo-to-thermal conversion of CuFe nanoparticles was investigated, potentially integrating with photodynamic treatment (PDT) for prospective advancements in the eradication of cancer cells following a single pulse of deep-red light irradiation at elevated laser intensity [93]. Table 1 summarize copper nanoformulations in cervical cancer.

Therefore, additional research and refinements are necessary to enhance effectiveness and evaluate their practicality in living organisms. Potential future research could involve improving the green synthesis method by exploring alternative biological materials, assessing the nanocomplexes in a co-culture setting with both standard and cancer cells to observe synergistic effects, and refining the receptor-mediated uptake in cervical cancer cells to improve transgene activity. Conducting mechanistic investigations to comprehend the cellular absorption and trafficking of the nanocomplexes in cells would be advantageous.

## 5. Cuproptosis, Cuproplasia, and Cancer

Activating intracellular copper overload is a potent and adaptable method for fighting cancer, and its underlying molecular mechanisms have been recently examined in detail. Researchers have introduced the word ‘cuproptosis’ to describe this specific form of cell death [1]. Cuproptosis is a newly identified kind of programmed cell death dependent on copper [94]. It can be activated by the effective transport of copper utilizing copper ionophores like elesclomol (ES) [95]. Tsvetkov’s research revealed that copper can induce the aggregation of lipid-acylated proteins and the depletion of iron–sulfur (Fe-S) cluster proteins [96].

Additionally, copper can enhance proteotoxic stress by directly binding to the lipid-acylated components of the tricarboxylic acid (TCA) cycle. Ultimately, these effects can result in cell death. Cuproptosis is a unique type of cell death distinct from other well-known forms, including pyroptosis, apoptosis, ferroptosis, and necroptosis [97]. Given the widespread and conservative presence of lipid acylation and Fe-S cluster proteins in nature, which are the primary targets of cytotoxicity caused by cuproptosis, there is promising potential for therapeutic options that target copper ions in tumors with this metabolic profile. However, the lack of reliable and precise biomarkers to detect cuproptosis in complex human tumor tissues is a significant challenge in cancer research. This is an area that requires urgent attention and further investigation.

Several molecular targets regulate the maintenance of copper levels in the human body. Ceruloplasmin (CP) is the primary protein carrier for delivering copper to organ and tissue systems [98]. The metalloreductases and CTR1 high-affinity Cu^+^ transporter work together at the cellular level to facilitate the effective absorption of copper. Subsequently, cytoplasmic and mitochondrial metallochaperones collaborate to facilitate the specific incorporation of copper into metalloprotein. ATP7A and ATP7B are responsible for both the copper export and metallochaperones’ functions [99].

**Figure 2:**
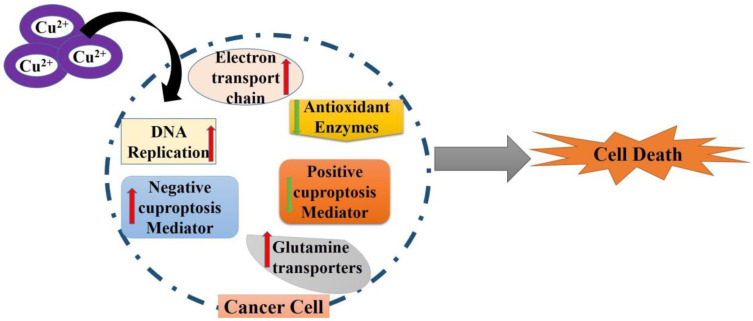
Copper induces cell death in cancer therapeutics. Increased copper concentration in cancer cells increases ETC, DNA replication, glutamine transporters, harmful cuproptosis mediators, and reduced levels of positive cuproptosis mediators and antioxidant enzymes. These alterations result in cell death.

In contrast to normal cells or tissues, cancer progression is typically associated with a significant rise in copper levels. A sufficiently elevated copper level is advantageous for sustaining cancer cells’ rapid growth and movement. Carcinogenesis is related to the disturbance of tumor suppressor genes, which are also referred to as antioncogenes [100]. Cells undergo neoplastic transformation when they develop the capacity to increase without control, defy programmed cell death, promote the growth of new blood vessels, and avoid detection by the immune system. Cancer is characterized as an uncontrolled growth of cells. Elevated copper concentrations have been identified in cancer cell lines, tissues, and the blood serum of patients [101]. Thus, there is a strong correlation between copper and the development of tumors. Cuproplasia, a recently introduced term by Ge et al., refers to the growth and proliferation of cells dependent on copper. This includes copper’s primary and secondary roles in tumor formation and proliferation, which occur through several signaling pathways [102]. This discovery opens up intriguing possibilities for cancer therapies. This encompasses both the fundamental and secondary functions of copper in terms of signaling pathways and copper-dependent cell development and proliferation. Cuproplasia has the potential to be a viable target for cancer therapies, while its involvement in tumor development requires further investigation. Activating cuproptosis with copper ionophores and suppressing cuproplasia through copper depletion are significant alternatives for anticancer treatment [103,104]. Therefore, cuproplasia plays a crucial role in cancer due to the rising need for copper in the development and spread of tumors. Cuproplasia is linked to multiple physiological functions, such as mitochondrial respiration, antioxidant defenses, redox signaling, kinase signaling, autophagy, and protein quality control [105]. Both copper chelators and copper ionophores can suppress tumor proliferation and enhance the efficacies of chemotherapy, immunotherapy, or radiation therapy [106]. They achieve this by reducing the accumulation of copper in cells (cuproplasia) and promoting cell death through copper-induced oxidative stress (cuproptosis), respectively [107]. Furthermore, clinical research has demonstrated higher copper levels in the serum of cancer patients or cancerous tissues compared to those in the standard control groups. A discovery in the field of copper-induced cell death is the study and creation of copper ionophores. Copper ionophores are molecules that facilitate the transport of copper from outside the cell to inside the cell. Copper ionophore compounds have recently seen significant improvements, making them a focal point in the research and development of future copper therapeutics (Figure 2).

### 5.1. Cuproptosis Related Genes

The following ten genes, FDX1, LIPT1, DLD, LIAS, DLAT, PDHA1, PDHB, MTF1, GLS, and CDKN2A, have been identified as genes associated with cuproptosis [108,109]. The FDX1 gene encodes a small iron–sulfur protein that facilitates electron transfer from NADPH to mitochondrial cytochrome P450 through ferredoxin reductase. This process involves the metabolism of steroids, vitamin D, and bile acids. FDX1 may impact the prognosis of lung adenocarcinoma by regulating metabolism [110]. Lipoyltransferase 1 (LIPT1) is an enzyme that catalyzes the activation of 2-ketoacid dehydrogenases linked with the tricarboxylic acid (TCA) cycle. Deficiency of LIPT1 hampers the metabolic activity of the TCA cycle [111]. The role of Dihydrolipamine dehydrogenase (DLD) in regulating ferroptosis triggered by cysteine deficiency has been documented. The occurrence of cuproptosis was suppressed by deleting FDX1 and LIAS. Increased DLAT expression can enhance gastric cancer cell growth via modulating glucose metabolism [112].

The abnormal expression of PDHA1 and PDHB, which play a role in regulating glycolysis, was strongly related to negative prognosis in gastric cancer patients. Metal regulatory transcription factor 1 (MTF1) is a metal-binding transcription factor in eukaryotes. It safeguards cells from oxidative and hypoxic stress by detecting both excessive and insufficient levels of metals [113]. Deletion of MTF1 could impede the epithelial-to-mesenchymal transition [114]. Glutaminase (GLS) is a crucial enzyme in the process of glutamine metabolism and has been found to play a role in several forms of cancer [115]. CDKN2A (tumor suppressor gene) is located on chromosome 9p21.3. It is involved in inhibiting the growth of tumors and preventing their proliferation. Nevertheless, the expression of CDKN2A was increased in hepatocellular carcinoma (HCC) and exhibited a significant correlation with unfavorable patient prognosis. Furthermore, there was a correlation between CDKN2A methylation levels and copper ion metabolism in individuals [116]. These findings indicate the participation of cuproptosis-related genes in the predictive progression of cancer.

### 5.2. Cuproptosis and lncRNAs (Long Non-Coding RNAs) in Cervical Cancer

**Figure 3:**
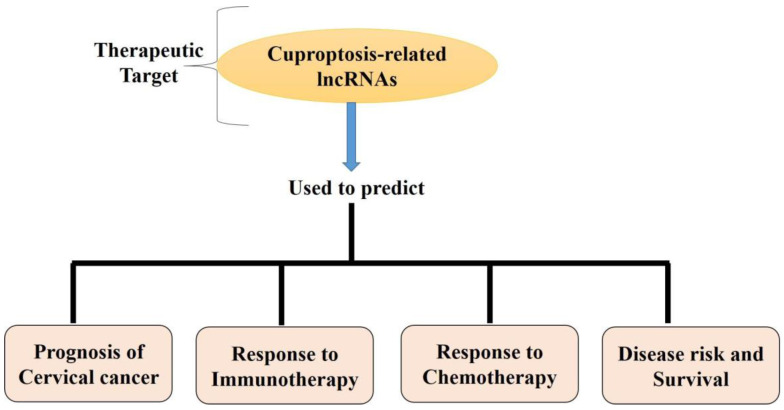
Cuproptosis-related lncRNAs’ role in cervical cancer.

lncRNAs have been implicated in various aspects of tumor development and advancement by regulating cancer cell activity [117]. Extensive research indicates that long non-coding RNAs (lncRNAs) play a role in the advancement, reoccurrence, and response to immunotherapy of cancer by controlling many epigenetic pathways, including hypoxia, m6A methylation, ferroptosis, autophagy, and energy metabolism [118,119,120]. More research needs to be conducted on the functions and roles of lncRNAs in cuproptosis, as studies are scarce. Understanding their contribution to the prognostic prediction of cancer patients is crucial. Long non-coding RNAs (lncRNAs) have been employed to construct a signature of lncRNAs linked with cuproptosis [121]. This signature can predict patient prognosis and response to immune checkpoint blockade (ICB) therapy [122]. Long non-coding RNAs (lncRNAs) were found to play a role in cuproptosis, a type of cell death (Figure 3). Researchers have created a predictive profile of lncRNAs associated with cuproptosis, which can further be utilized to predict cancer patients’ response to immunotherapy. This discovery opens up new possibilities for non-apoptotic therapeutic approaches in cancer treatment.

In their study, Huang et al. (2024) identified and described five lncRNA predictive models associated with cuprotosis. These models can potentially serve as novel therapeutic targets for cervical cancer treatment and prevention. These findings provide an essential basis and point of reference for further investigating the causes and mechanisms of cuproptosis and devising more efficient patient therapies. When evaluating the prognosis and therapy options for cuproptosis, physicians and scientists must also consider these genes’ effects [123].

A prognostic signature was created using seven long non-coding RNAs (lncRNAs) associated with cuproptosis. This signature includes five lncRNAs (AL441992.1, LINC01305, AL354833.2, CNNM3-DT, and SCAT2) that have a protective effect and two lncRNAs (AL354733.3 and AC009902.2) that pose a danger. A prognostic risk signature of long non-coding RNA (lncRNA) associated with cuproptosis was developed to predict prognosis in patients with CC. In addition, the signature can anticipate the response to immunotherapy and chemotherapy, enabling clinicians to develop tailored treatment strategies for patients with CC [124]. A novel study devised a distinctive risk model involving six long non-coding RNAs (lncRNAs) associated with cuproptosis, which could potentially serve as a predictive tool for patients with cervical cancer. An assessment was conducted on the effectiveness of immune checkpoint inhibitors and chemosensitivity.

The relationship between the risk model and the immunological environment was also examined. These observations could potentially enhance the effectiveness of immune therapy-based therapies and chemotherapy in individuals diagnosed with cervical cancer. To account for genetic heterogeneity, evaluating these lncRNAs in additional public databases is necessary. Furthermore, obtaining conclusive conclusions would require a bigger sample size, and the prediction model should be practically confirmed before its application in clinical patients [125].

Further investigations were conducted to investigate the correlation between the expression of cuproptosis-related long non-coding RNAs (lncRNAs) and the stage and prognosis of cervical cancer. A novel index, called the cuproptosis-related lncRNA risk score (CRL risk score), was introduced to assess the risk of cuproptosis and determine the survival status based on the association with lncRNAs [126]. An analysis was conducted on the gene mutation pattern, immune-related components, and sensitivity to chemotherapeutic medications of cervical cancer patients in distinct risk groups. The results revealed significant disparities in these features between the two risk categories. The predictive-type model has considerable potential in guiding the typing and treatment of cervical cancer in patients with CRL (cuproptosis-related lncRNA) risk score sensitivity. It has been demonstrated to have therapeutic value and relevance [127]. A prognostic signature of three long non-coding RNAs (lncRNAs) associated with cuproptosis has been discovered. This signature has been demonstrated to be both independent and highly dependable. Our work investigated and identified prospective biomarkers and therapeutic targets for cuproptosis-related signatures in cervical cancer. This finding has significant clinical implications, such as enhancing the accuracy of predicting cervical cancer in patients and offering a biomarker for cervical cancer [128].

Alternative splicing has become a prominent area of study in cancer research in recent years [129]. Therefore, the combination of alternative splicing, pyroptosis, and cuproptosis is precious for investigating their combined influence on the development and advancement of cervical cancer. This work suggests that the alternate splicing of genes linked with pyroptosis and cuproptosis is crucial in reshaping the phenotypic characteristics of the cervical cancer tumor microenvironment (TME) via influencing immune responses and metabolic pathways. This study offers significant insights into the interaction between alternative splicing variants associated with pyroptosis, cuproptosis, and the tumor microenvironment (TME). These findings enhance our understanding of the development of cervical cancer and prospective treatment options [130].

Cuproptosis and tumor angiogenesis have a strong correlation in the tumor microenvironment [131]. The CuRA genes may elucidate the possible association between cuproptosis and angiogenesis, enhancing the prognosis of individuals with cervical cancer [132]. Therapeutic approaches that focus on inhibiting the growth of blood vessels (angiogenesis) have been employed in the medical treatment of individuals who have advanced or recurring cervical cancer [133]. Cuproptosis is a significant factor in the growth and development of tumor cells and the formation of new blood vessels. Analysis of cell–cell communication using single-cell sequencing provides insights into the tumor immunological microenvironment and alterations within the tumor. A study by Kang et al. (2024) utilized single-cell RNA sequencing and bulk RNA sequencing data to develop a gene signature called CuRA associated with cuproptosis-related angiogenesis [132]. This gene signature shows promise in predicting outcomes for patients with cervical cancer [132]. However, further validation is required through in vivo and in vitro experiments.

Cuproptosis mainly occurs when lipoylated components of the tricarboxylic acid (TCA) cycle aggregate due to copper binding [134]. This process also leads to the instability of Fe-S cluster proteins in mitochondria. Up to now, various copper transporters, including dithiocarbamates (DTCs), thiosemicarbazones (TSCs), hydroxyquinolines (HQs), hydroxyflavones (HFs), and curcumin (Cur), have been utilized to eliminate cancer cells by delivering copper inside them [135]. The study employed proteomics to thoroughly examine the interaction between copper stress and cuproptosis in cancer cells. The authors further investigated the potential of curcumin as a biologically active copper transporter for cancer treatment. A new compound called [1-propyl-3,5-bis(2-bromobenzylidene)-4-piperidinone] (PBPD) was developed to enhance both the absorption into the body and the effectiveness against tumors. PBPD suppresses the acidity of the tumor microenvironment and decreases cellular metabolism, hence impeding the invasion and migration of cervical cancer cells [136]. The study by Zhang et al. in 2024 found that PBPD effectively suppresses cervical cancer cells’ growth, infiltration, and movement. These findings suggest that copper has the potential to be a promising new drug conjugate for treating cervical cancer.

## 6. Conclusions

Copper triggers tumor cell death through many mechanisms, and “cuproptosis” is a newly discovered regulated cell death different from apoptosis, iron death, autophagy, and planned necrosis. Following a sequence of rigorous safety and efficacy examinations can facilitate the translation of fundamental chemical and biological investigations of copper into possible clinical treatments for cervical cancer. Moreover, it exhibits encouraging prospects in the realm of tumor therapy. The precise processes through which cuproptosis operates in cancer remain unknown, and a substantial body of rigorous foundational research is required to establish a cause-and-effect relationship between cuproptosis and malignancies. The identification of cuproptosis has introduced novel prospects for the treatment of cervical cancer. Nevertheless, additional investigation is vital for implementing this method in clinical settings.

## Figures and Tables

**Table 1:** Copper nanoformulations targeting cervical cancer cells.

Copper Nanoparticles	Components of Nanoformulation	Mode of Action	References
Hc-CuONPs	Copper + plant extract of H. cordata	Apoptosis induction	[75]
		Altered PI3k/AKT/mTOR signaling	
		Enhanced expression of pro-apoptotic proteins	
		Reduced expression of anti-apoptotic protein	
Mixed-ligand copper(II) complex	Cu(L)(phen)]⋅MeOH (L = 4-chloro-2-[(2-hydroxyphenyl)iminomethyl]phenol)	Apoptosis induction	[76]
		Cell cycle arrest	
		Induced DNA fragmentation	
		Increased generation of reactive oxygen species	
Cu_4_O_3_ NPs	Cu_4_O_3_ + aqueous extract of pumpkin seeds	Increased generation of reactive oxygen species	[78]
		MMP modulation	
		Suppressed cell adhesion/migration	
CuONPs	CuO + Melia azedarach leaf extract + chitosan + polyethylene glycol (PEG) + ligand folate	Binding and protection of the reporter gene	[85]
		Reduced cell viability	
		Significant transgene expression	
		Efficient gene delivery	
Cu/TiO_2_	Copper complexes coupled to TiO_2_ nanoparticles	Increased cytotoxic potential	[88]
		Increased cell death	
CuL(SCN)	Single crystal of the copper-complex + NaSCN	Increased cytotoxicity to cancer cells	[90]
		ROS accumulation	
		Initiation of nuclear blebbing	
		DNA degradation	
CuO NPs	CuO + aqueous black bean extract	Increased ROS generation	[92]
		Altered mitochondrial structure	
		Initiated lipid peroxidation of the liposomal membrane	
		Suppressed cell proliferation	
		Apoptosis induction	

## Data Availability

No data were used for the research described in the article.

## Abbreviations

Cu	Copper
ROS	Reactive oxygen species
DTCs	Dithiocarbamates
TSCs	Thiosemicarbazones
HFs	Hydroxyflavones
Cur	Curcumin
HQs	Hydroxyquinolines
TCA	Tricarboxylic acid
PBPD	[1-propyl-3,5-bis(2-bromobenzylidene)-4-piperidinone]
TME	Tumor microenvironment
lncRNAs	Long non-coding RNAs
CRLs	Cuproptosis-related lncRNAs
MTF1	Metal regulatory transcription factor 1
LIPT1	Lipoyltransferase 1
SLC31A1	Solute carrier family 31 member 1
ETC	Electron transport chain
ICB	Immune checkpoint blockade
